# Supplementary figures and images for: Control of clathrin-mediated endocytosis by NIMA family kinases
Source: PLoS Genet. 2020 Feb 18;16(2):e1008633. doi: 10.1371/journal.pgen.1008633 (PMC7048319; doi:10.1371/journal.pgen.1008633)

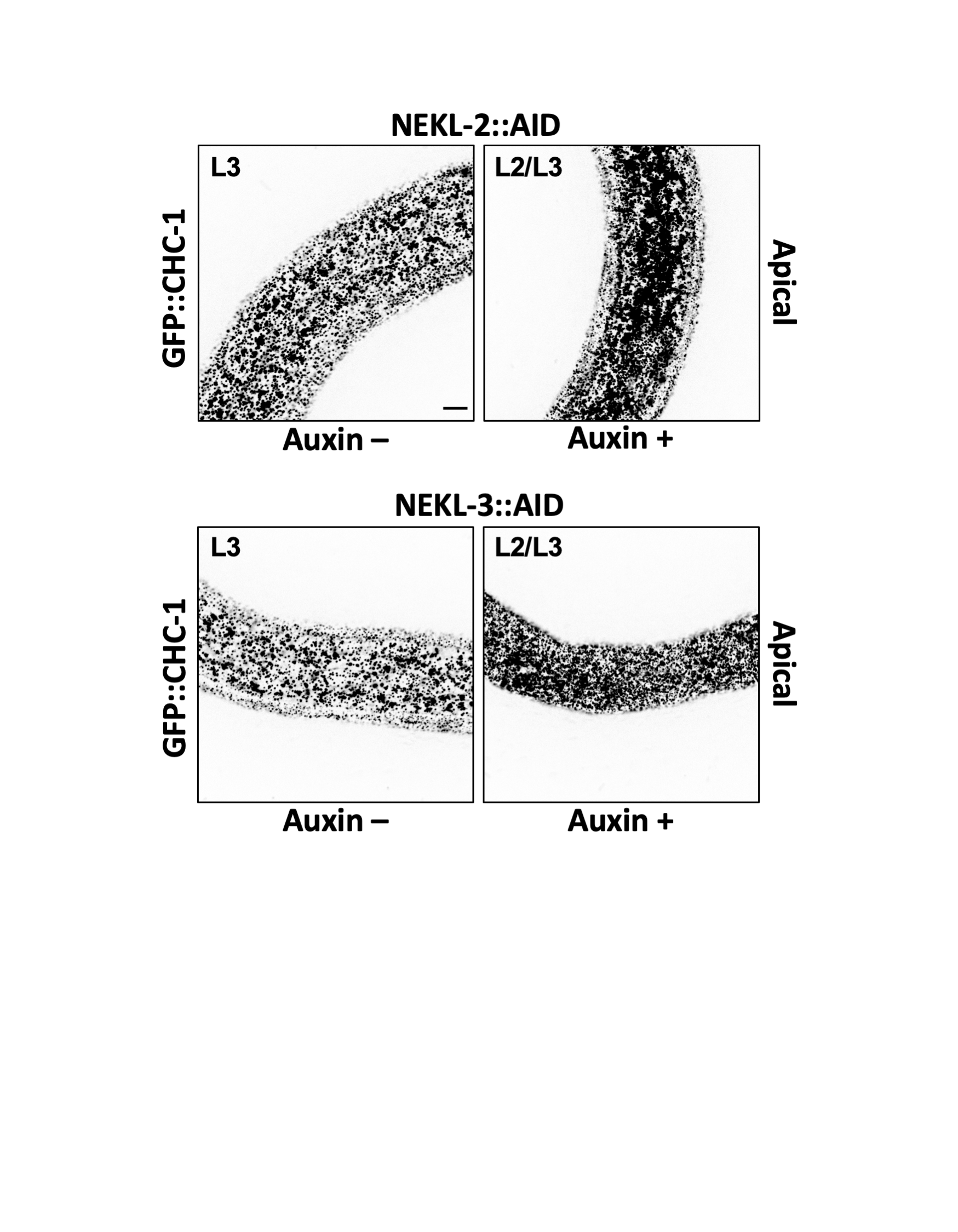

Supplement: S1 Fig — Representative images of untreated (Auxin–) and auxin-treated (Auxin +; 20 h) NEKL-2::AID and NEKL-3::AID arrested larvae expressing GFP::CHC-1. Inverted fluorescence images are shown to aid clarity. Background subtraction was performed using the same parameters for all images; minimum and maximum pixel values were kept consistent for all images. Bar in upper left panel = 5 μm (for all panels). (TIFF) [file pgen.1008633.s001.tiff]

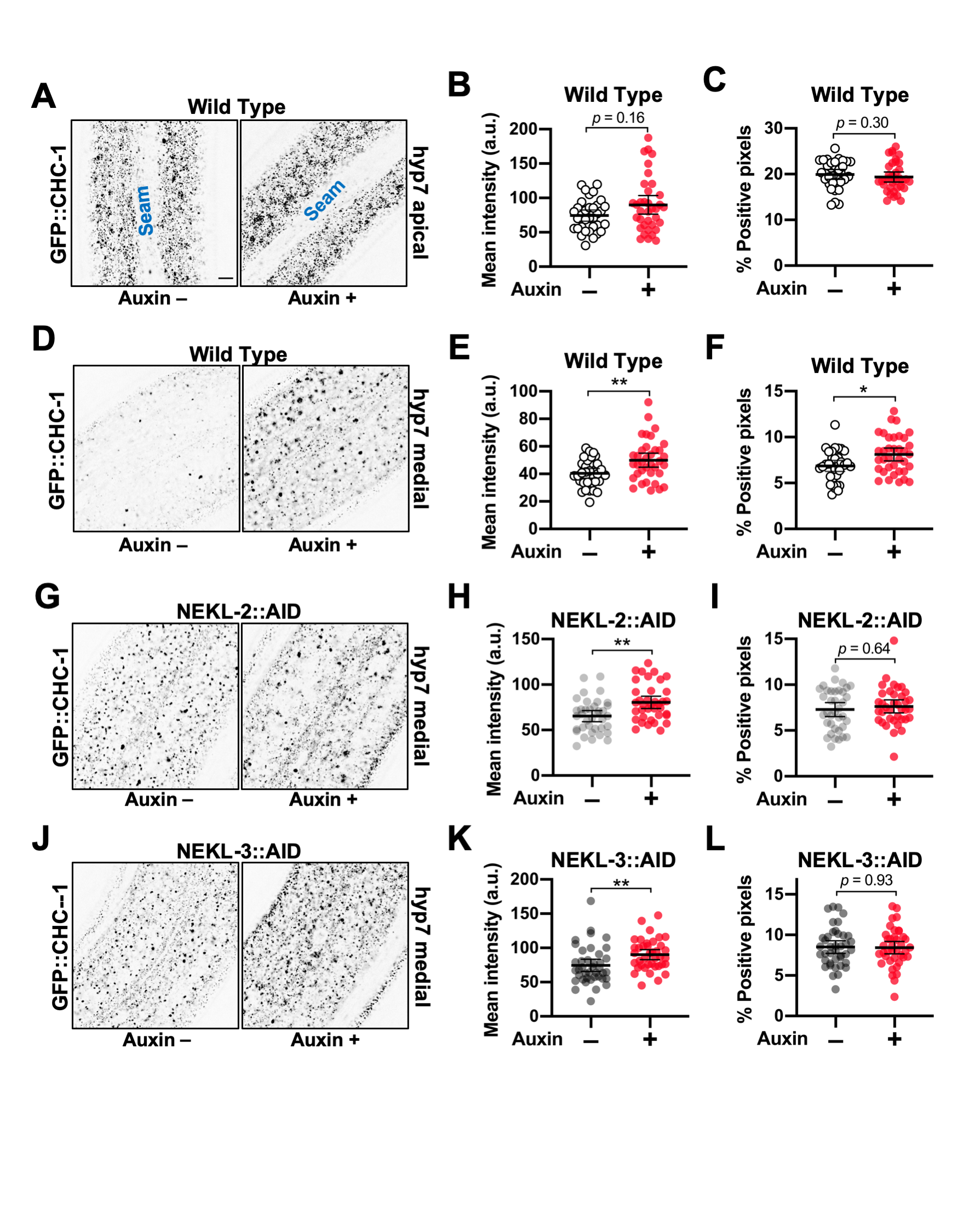

Supplement: S2 Fig — (A–F) Representative images of untreated (Auxin–) and auxin-treated (Auxin +; 20 h) wild-type day-2 adults expressing GFP::CHC-1. (G–L) Representative images of similarly treated NEKL-2::AID (G–I) and NEKL-3::AID (J–L) day-2 adults expressing GFP::CHC-1. Inverted fluorescence was used to aid clarity. Background subtraction was performed using the same parameters for all images; minimum and maximum pixel values were kept consistent for all images. Bar in A = 5 μm (for all panels). Mean GFP::CHC-1 intensities (B,E,H,K) and the percentage of GFP-positive pixels above threshold (C,F,I,L) were determined for day-2 adults. Panels A–C show data for the apical region of hyp7; panels D–L show data for a medial region of hyp7. (B,C,E,F,H,I,K,L) Both the group mean and 95% confidence interval (error bars) are shown. Note that wild-type showed small but statically significant increases in medial GFP::CHC-1 intensity and pixels above threshold after exposure to auxin, suggesting that auxin itself could exert a weak effect on GFP::CHC-1 localization. p-Values were determined using two-tailed Mann-Whitney tests; **p < 0.01, *p < 0.05. Raw data are available in S1 File. (TIFF) [file pgen.1008633.s002.tiff]

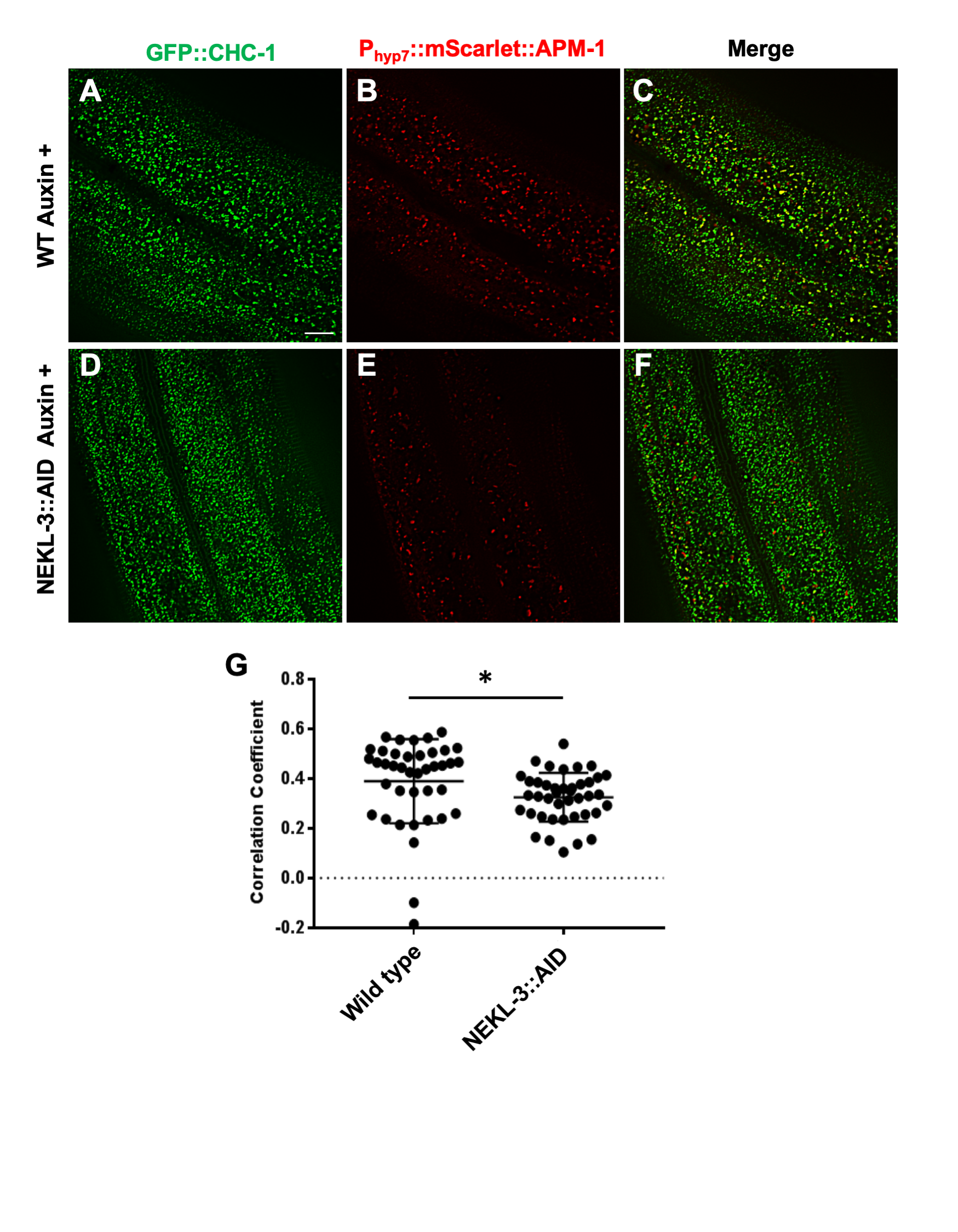

Supplement: S3 Fig — (A–F) Representative images of (A,D) GFP::CHC-1, (B,E) PY37A1B.5APM-1::mScarlet, and merged images (C,F) in auxin-treated (A–C) wild-type and (D–F) NEKL-3::AID adults. Bar size in A = 5 μm (for A–F). (G) Pearson’s r coefficients are shown for the indicated strains; circles correspond to images from individual worms. (H,I) p-Values were determined using student’s T-Test; *p < 0.05. Raw data are available in S1 File. (TIFF) [file pgen.1008633.s003.tiff]

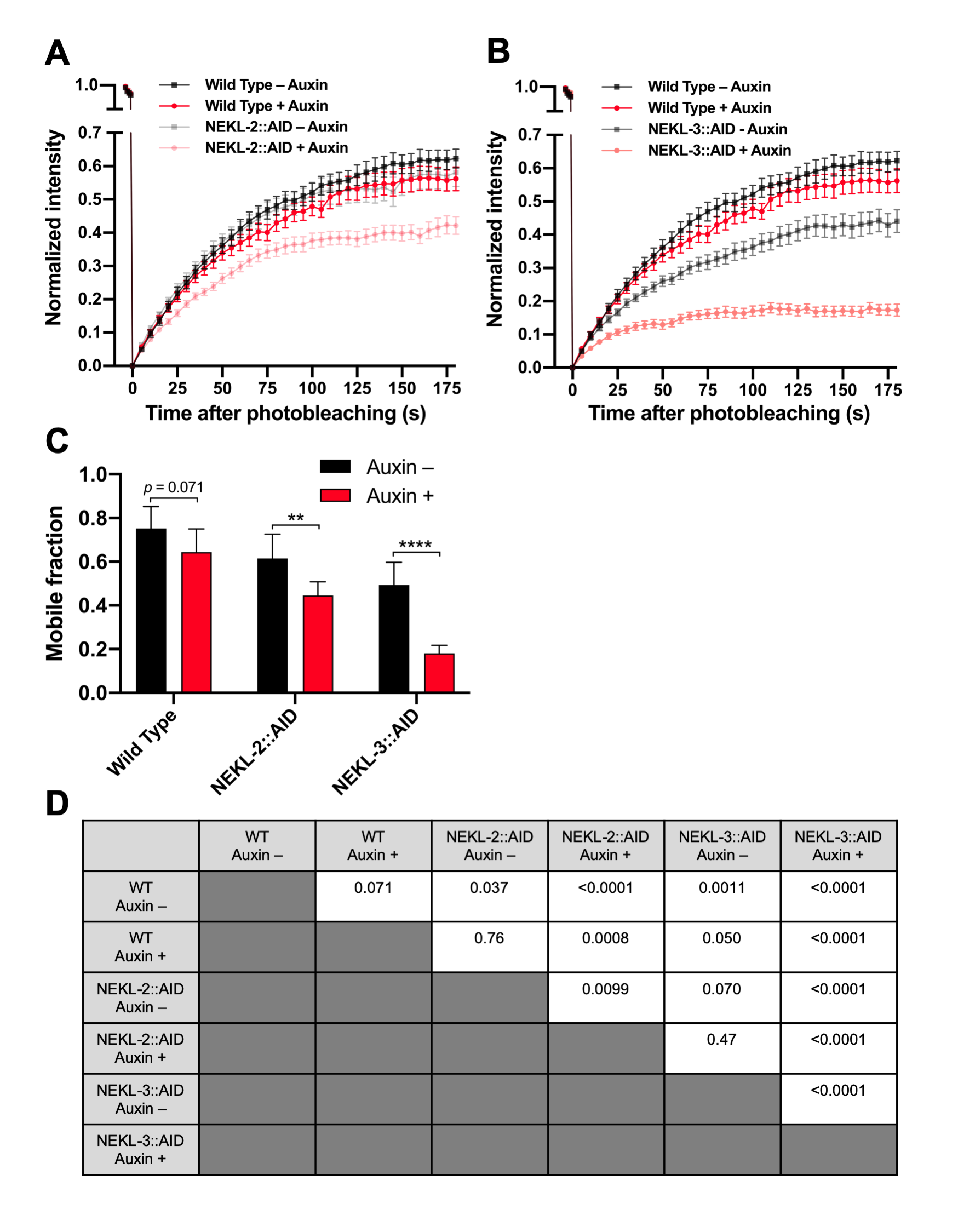

Supplement: S4 Fig — (A,B) Fluorescence recovery curves for wild-type (A,B), NEKL-2::AID (A), and NEKL-3::AID (B) day-2 adults in the presence and absence of auxin (20 h). Analyses were carried in the apical hyp7 region with GFP::CHC-1. Normalized average mean intensities of the photobleached regions were plotted as a function of time using 5-s intervals; error bars denote SEM. (C) Mobile fractions from FRAP data in panels A and B; error bars show 95% confidence intervals. (D) p-Values for all possible comparisons for data in panel C were determined using two-tailed Mann-Whitney tests. Raw data are available in S1 File. (TIFF) [file pgen.1008633.s004.tiff]

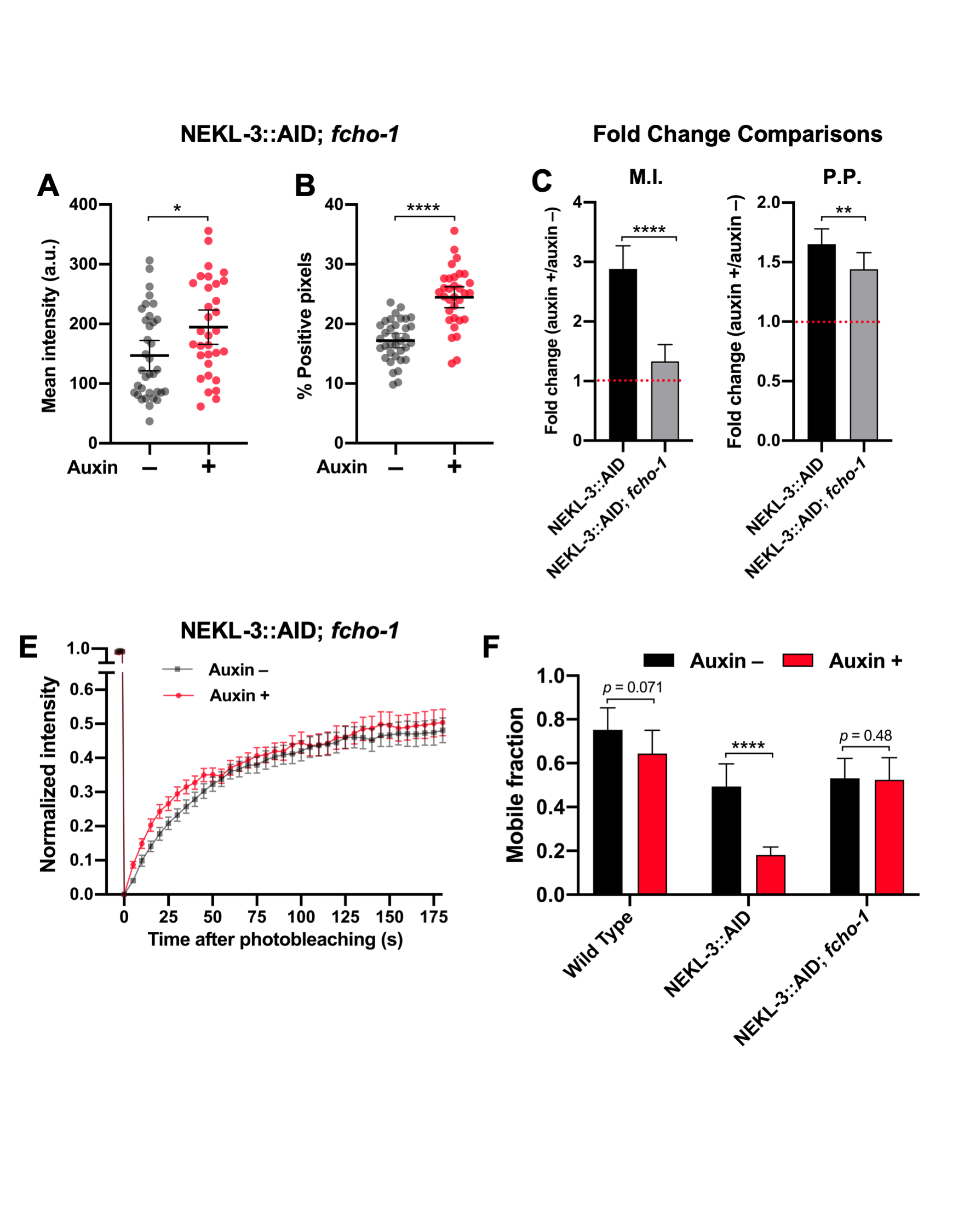

Supplement: S5 Fig — Mean GFP::CHC-1 intensities (A) and the percentage of GFP-positive pixels above threshold (B) were determined for individual adults. (C) Comparative fold changes for mean intensities (M.I.) and positive pixels above threshold (P.P) are shown for the indicated genotypes in the presence (+) and absence (–) of auxin. (D) Fluorescence recovery curves of NEKL-3::AID fcho-1(fd296) day-2 adults in the presence and absence of auxin. Normalized average mean intensities of photobleached regions were plotted as a function of time using 5-s intervals; error bars denote SEM. (E) Bar plot showing the mobile fractions from FRAP analyses of wild-type, NEKL-3::AID, and NEKL-3::AID fcho-1(fd296) adults. (C,F) Error bars show 95% confidence intervals. The dashed red line at 1.0 indicates no change in auxin-treated versus untreated worms. Statistical analyses for ratios (C) were carried out as described in the Materials and Methods; **p < 0.01, ****p < 0.0001. Raw data are available in S1 File. (TIFF) [file pgen.1008633.s005.tiff]

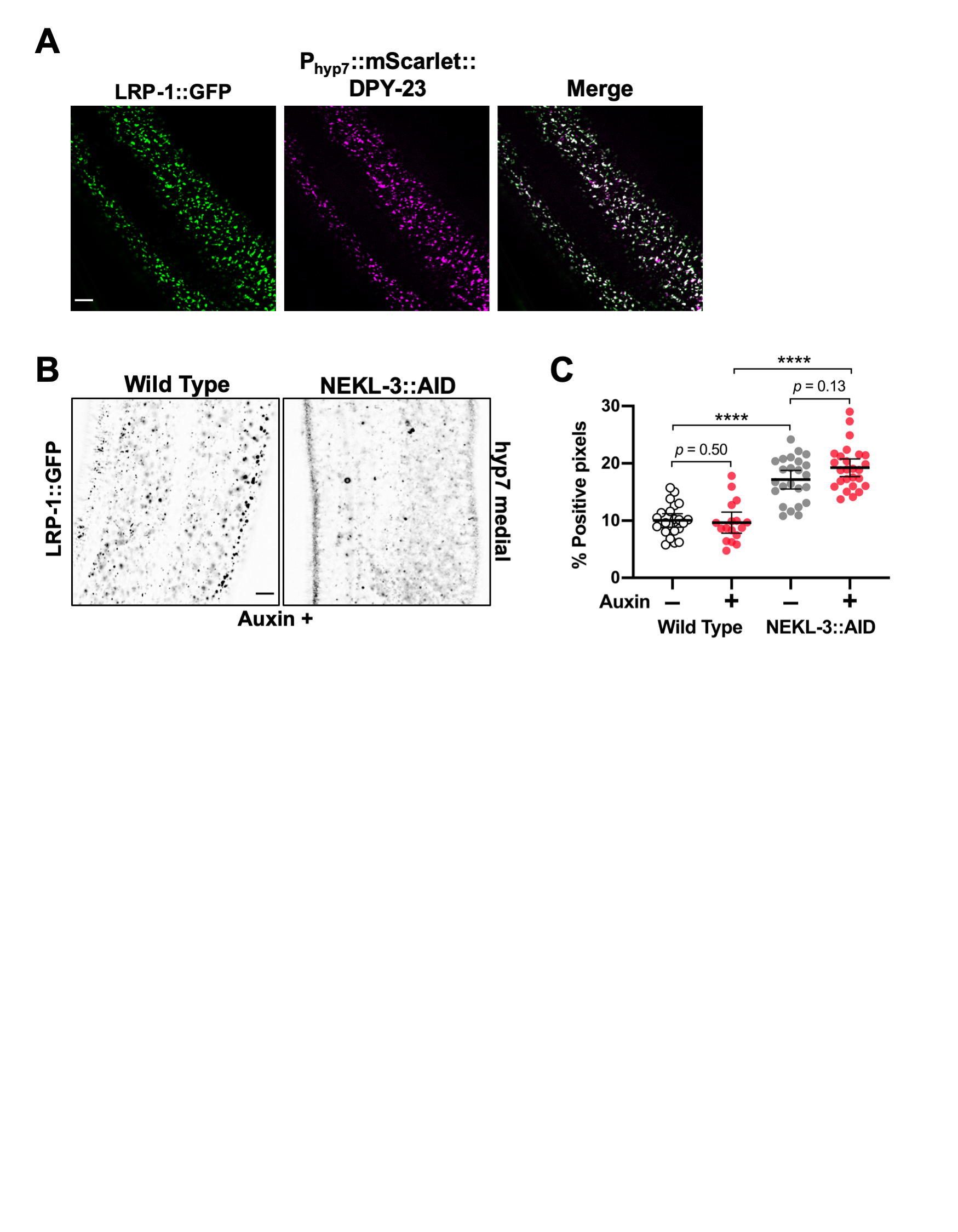

Supplement: S6 Fig — (A) Representative images showing strong colocalization of LRP-1::GFP and a marker for AP2, Pdpy-7::mScarlet::DPY-23. mScarlet is represented as magenta, overlap is white. (B) Representative confocal images of LRP-1::GFP in auxin treated wild-type and NEKL-3::AID adults. Images show the medial plane of hyp7 (also see S13 and S14 Movies). Inverted fluorescence was used to aid clarity. Bar size in A,B = 5 μm. (C) Percentage of GFP-positive pixels above threshold were determined for individual adults. p-Values were determined using two-tailed Mann-Whitney tests; ****p < 0.0001. Raw data are available in S1 File. (TIFF) [file pgen.1008633.s006.tiff]

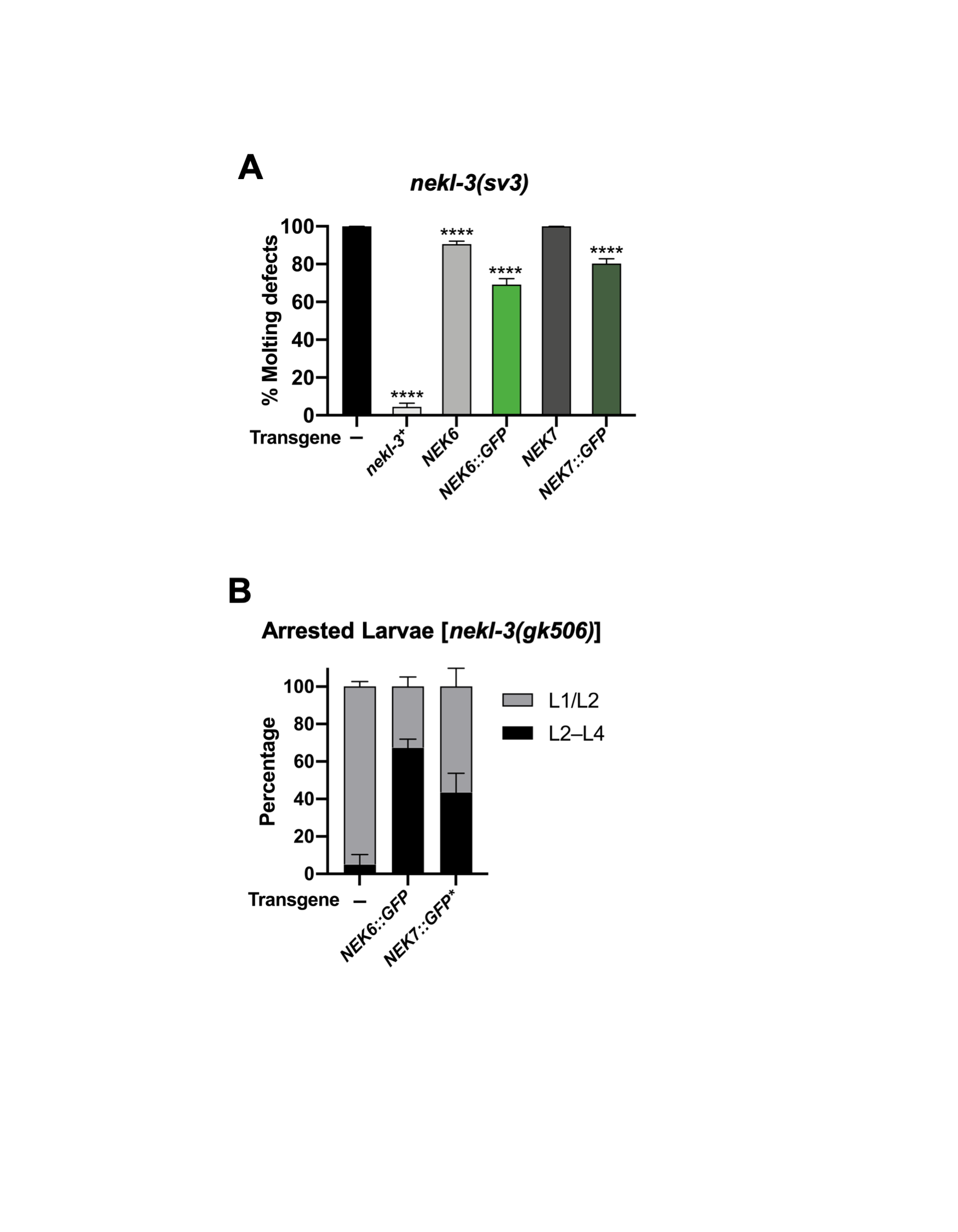

Supplement: S7 Fig — (A,B) Bar plot showing rescue of molting defects in nekl-3(sv3) strains with the indicated transgenes. NEK6::GFP and NEK7::GFP refer to Pnekl-3::NEK6::GFP and Pnekl-3::NEK7::GFP, respectively. NEK6 and NEK7 refer to Pnekl-3::NEK6 and Pnekl-3::NEK7, respectively. p-Values were determined using Fischer’s exact test; ****p < 0.0001. (B) Bar plot showing the percentage of L1/L2 versus L2–L4 arrested larvae in transgene-positive nekl-3(gk506) mutants. Note that ~40–60% of transgene-positive arrested larvae bypass the L1/L2 arrest point, whereas the large majority of transgene-minus worms arrest at L1/L2. Given ~50% rescue to adulthood by the NEK6::GFP and NEK7::GFP transgenes (Fig 12A), partial-to-full rescue occurs at a frequency of ~75% in transgene-positive nekl-3(gk506) mutants. (TIFF) [file pgen.1008633.s007.tiff]

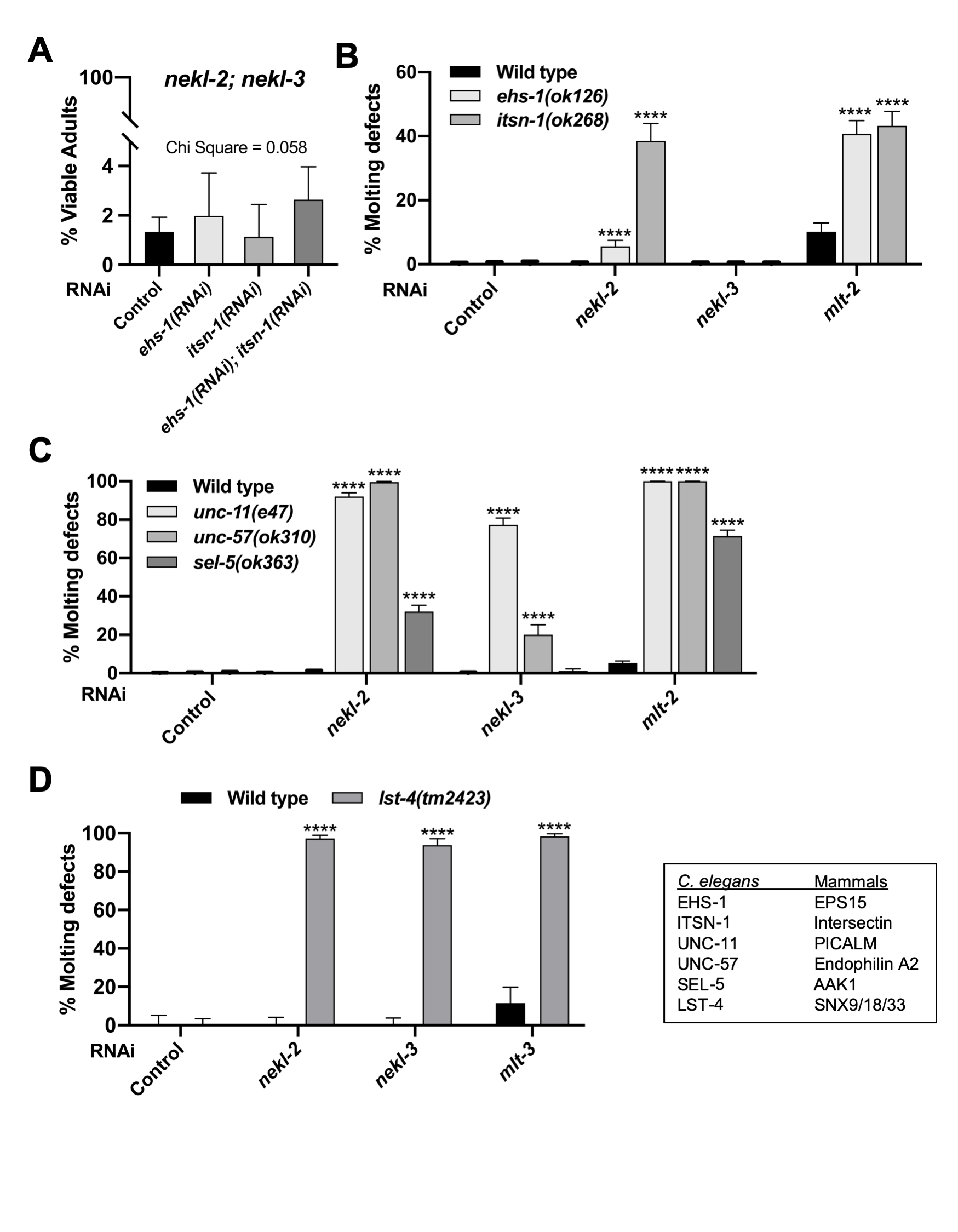

Supplement: S8 Fig — (A) Bar plot showing failure to suppress molting defects in nekl-2(fd81); nekl-3(gk894345) double mutants by RNAi of ehs-1, itsn-1, and ehs-1; itsn-1, using dsRNA injection methods. (B–E) RNAi feeding of the nekl-2, nekl-3, and mlt-2 was carried out in the indicated backgrounds. Error bars indicate 95% confidence intervals; p-values were determined using Fischer’s exact test where proportions were compared to the wild-type allele. ****p < 0.0001, Raw data are available in S1 File. (TIFF) [file pgen.1008633.s008.tiff]
