# Supplementary material for: Control of clathrin-mediated endocytosis by NIMA family kinases
Source: PLoS Genet. 2020 Feb 18;16(2):e1008633. doi: 10.1371/journal.pgen.1008633 (PMC7048319; doi:10.1371/journal.pgen.1008633)
Supplement: S2 Text — (DOCX) [file pgen.1008633.s011.docx]

**S2 Text.** Authorship contributions by figure.

Fig 1. Braveen Joseph carried out genetic screening, cloning, and genetic analysis; Phil Edeen aided with CRISPR and RNAi studies; David Fay aided with genetic analysis, CRISPR, and RNAi.

Fig 2. Braveen Joseph carried out genetic screening, cloning, and genetic analysis; Phil Edeen aided with CRISPR and RNAi studies; David Fay aided with genetic analysis, CRISPR, and RNAi.

Fig 3. Braveen Joseph carried out strain construction and genetic analysis.

Fig 4. Braveen Joseph carried out strain construction and genetic analysis. Phil Edeen aided with CRISPR and RNAi studies.

Fig 5. Barth Grant generated the clathrin GFP reporter (Figs 5–9, 11, 12, S1­–5). Braveen Joseph carried out strain construction, imaging, and quantification.

Fig 6. Yu Wang generated the NEKL::AID strains (Figs 6–10, 12, S1–S6 ), carried out western blots (Fig 6B), and made the initial observation that clathrin was altered in NEKL::AID strains; Braveen Joseph carried out the depletion studies, imaging, and quantification (6C–K).

f

Fig 7. Yu Wang generated the mScarlet::DPY-23 reporter (Fig 7, S6), carried out strain construction (Fig 7A–F), and independently repeated and verified the results shown in Fig 7A-G; Braveen Joseph aided with strain construction (Fig 7A–F) and carried out the imaging and quantification (Fig 7A–I).

Fig 8. Braveen Joseph did the imaging and quantification.

Fig 9. Braveen Joseph did the strain construction, imaging, and quantification; Phil Edeen aided with CRISPR experiments.

Fig 10. Braveen Joseph did the strain construction, imaging, and quantification.

Fig 11. Braveen Joseph did the strain construction, imaging, and quantification.

Fig 12. Braveen Joseph did the imaging, quantification, rescue studies, and aided with plasmid construction; Vladimir Lazertic generated the NEK6/7::GFP constructs and made the initial observation of rescue; Phil Edeen aided with plasmid and strain construction; David Fay aided with rescue analyses.

S1 Fig. Braveen Joseph did the imaging.

S2 Fig. Braveen Joseph did the imaging and quantification.

S3 Fig. Yu Wang generated the mScarlet::APM-1 reporter, carried out strain construction, and performed the imaging and quantification.

S4 Fig. Braveen Joseph did the imaging and quantification;

S5 Fig. Braveen Joseph did the imaging and quantification.

S7. Fig. Braveen Joseph did the imaging and quantification; Phil Edeen aided with strain construction.

S8. Fig. Braveen Joseph did the genetic analysis.
