# Supplementary material for: Control of clathrin-mediated endocytosis by NIMA family kinases
Source: PLoS Genet. 2020 Feb 18;16(2):e1008633. doi: 10.1371/journal.pgen.1008633 (PMC7048319; doi:10.1371/journal.pgen.1008633)
Supplement: S2 Table — (DOCX) [file pgen.1008633.s013.docx]

| **Strain** | **Genotype** |
| --- | --- |
| CB47 | *unc-11(e47)* |
| EG2710 | *unc-57(ok310)* |
| EG6353 | *fcho-1(ox477::unc-119(+); unc-119(ed3))* |
| GUN86 | *ncap-1(mew39)* |
| LH191 | eqIs1(lrp-1::gfp); rrf-3(pk1426) |
| LH373 | nekl-3(gk506); mnEx174(F19H6; pTG96) |
| NM1568 | *ehs-1(ok146)* |
| RB638 | *sel-5(ok363)* |
| RT3402 | *pw17(gfp::chc-1)* |
| RT3607 | *pw27(nekl-2::aid); ieSi57(peft-3::mRuby::tir-1); pw17(gfp::chc-1)* |
| RT3608 | *ieSi57(peft-3::mRuby::tir-1); pw17(gfp::chc-1); pw29(nekl-3::aid)* |
| RT3669 | *pw29(nekl-3::aid); pwSi10(phyp-7::bfp::tir-1); pw17(gfp::chc-1); pwSi30 (phyp-7::apm-1::mScarlet)* |
| RT3671 | *pw29(nekl-3::aid); pwSi10(phyp-7::bfp::tir-1); pw17(gfp::chc-1); pwSi44 (phyp-7::dpy-23::mScarlet)* |
| SP2734 | *mlt-4(sv9); mnEX173(mlt-4(+); pTG96)* |
| SP2736 | nekl-3(sv3); mnEx174(F19H6; pTG96) |
| VC201 | *Itsn-1(ok268)* |
| WY1061 | nekl-2(gk839); fdEx257 |
| WY1098 | *mlt-3(fd72); fdEx267(WRM0601dD02; pTG96(sur-5::GFP))* |
| WY1145 | nekl-2(fd81); nekl-3(gk894345); fdEx286 *(pDF153(*nekl-3 (+)); *pTG96(sur-5::GFP))* |
| WY1155 | *nekl-2(fd90[Y84L,G87A,G88A]; fdEx278)* |
| WY1165 | *nekl-2(fd91[Y84L,G87A]; fdEx278)* |
| WY1209 | *nekl-2(fd81); fcho-1(fd131); nekl-3(gk894345)* |
| WY1271 | *nekl-2(fd81); dpy-23(fd155) nekl-3(gk894345)* |
| WY1350 | *nekl-2(fd81); fcho-1(fd211); nekl-3(gk894345)* |
| WY1351 | *nekl-2(fd81); fcho-1(fd212); nekl-3(gk894345)* |
| WY1401 | *nekl-3(sv3); fdEx316(*pDF241*(NEK6::gfp(+));* pTG96.2*)* |
| WY1405 | *nekl-3(sv3); fdEx317(*pDF245*(NEK7::gfp(+));* pTG96.2*)* |
| WY1470 | *pw17(gfp::chc-1); pwSi30(phyp-7::apm-1::mScarlet)* |
| WY1471 | *pw17(gfp::chc-1); pwSi44(phyp-7::dpy-23::mScarlet)* |
| WY1474 | *dpy-23(mew74)* |
| WY1480 | *dpy-23(mew25)* |
| WY1509 | *pw17(gfp::chc-1); dpy-23(mew74)* |
| WY1510 | *ncap-1(mew39); pw17(gfp::chc-1)* |
| WY1531 | *dpy-23(fd261)* |
| WY1532 | *nekl-2(fd81); pw17(gfp::chc-1); nekl-3(gk894345);* fdEx286 *(pDF153(*nekl-3 (+)); *pTG96(sur-5::GFP))* |
| WY1533 | *ncap-1(mew39); pw17(gfp::chc-1); dpy-23(mew74)* |
| WY1534 | *nekl-2(fd81); fcho-1(fd262); nekl-3(gk894345)* |
| WY1538 | *nekl-2(fd90); fcho-1(ox477::unc-119(+))* |
| WY1539 | *ieSi57(peft-3::mRuby::tir-1); pw17(gfp::chc-1); pw29(nekl-3::aid); fdEx327(pDF421(NEK6(+)); pTG96.2)* |
| WY1550 | *nekl-2(fd81); fcho-1(fd131); pw17(gfp::chc-1); nekl-3(gk894345)* |
| WY1551 | *nekl-2(fd81); pw17(gfp::chc-1); dpy-23(fd155) nekl-3(gk894345)* |
| WY1560 | *nekl-2(fd81); dpy-23(fd277) nekl-3(gk894345)* |
| WY1562 | eqIs1(lrp-1::gfp); *ieSi57(peft-3::mRuby::tir-1); pw29(nekl-3::aid)* |
| WY1563 | *ieSi57(peft-3::mRuby::tir-1); pw17(gfp::chc-1); pw29(nekl-3::aid); fdEx330(*pDF153*(nekl-3(+));* pTG96.2*)* |
| WY1569 | *nekl-2(gk839); fcho-1(ox477::unc-119(+)* |
| WY1570 | *fcho-1(ox477::unc-119(+))*; *nekl-3(gk506)* |
| WY1571 | *fcho-1(ox477::unc-119(+)); mlt-3(fd72)* |
| WY1572 | *nekl-2(fd81); dpy-23(fd279) nekl-3(gk894345)* |
| WY1573 | eqIs1(lrp-1::gfp); *ncap-1(mew39)* |
| WY1574 | eqIs1(lrp-1::gfp); *dpy-23 (mew74)* |
| WY1575 | *nekl-2(fd81); apa-2(fd280) nekl-3(gk894345)* |
| WY1576 | *nekl-2(fd81); apa-2(fd281) nekl-3(gk894345)* |
| WY1577 | *apa-2(fd282)* |
| WY1578 | *pw27(nekl-2::aid); ieSi57(peft-3::mRuby::tir-1); pw17(gfp::chc-1); apa-2(fd283)* |
| WY1580 | *apa-2(fd285) nekl-3(gk506);* *mnEx174* *(F19H6; pTG96)* |
| WY1592 | *nekl-2(fd81); fcho-1(ox477::unc-119(+)); nekl-3(gk894345)* |
| WY1593 | *pw27(nekl-2::aid); fcho-1(fd296) ieSi57(peft-3::mRuby::tir-1); pw17(gfp::chc-1)* |
| WY1596 | *nekl-3(gk506); fdEx337(pDF422(NEK7(+)); pTG96.2);* mnEx174(F19H6; pTG96) |
| WY1598 | *ieSi57(peft-3::mRuby::tir-1); pw17(gfp::chc-1); pw29(nekl-3::aid); fdEx339(pDF422(NEK7(+)); pTG96.2)* |
| WY1602 | *nekl-3(gk506); fdEx343(pDF421(NEK6(+)); pTG96.2)* |
| WY1608 | *nekl-3(sv3); fdEx351(pDF421(NEK6(+)); pTG96.2)* |
| WY1609 | *nekl-3(gk506); fdEx345(pDF241(NEK6::gfp(+)); pTG96.2)* |
| WY1612 | *nekl-3(gk506); fdEx348(pDF244(NEK7::gfp(+)); pTG96.2)* |
| WY1615 | *nekl-3(sv3); fdEx351(pDF422(NEK7(+)); pTG96.2);* mnEx174(F19H6; pTG96) |
| WY1634 | eqIs1(lrp-1::gfp); *pwSi44(phyp-7::dpy-23::mScarlet)* |
| WY1636 | *fcho-1(ox477::unc-119(+) ieSi57(peft-3::mRuby::tir-1); pw17(gfp::chc-1); pw29(nekl-3::aid)* |
| WY1649 | lst-4(tm2423) |

**S2 Table. List of strains used in this study.**
