# Supplementary material for: Control of clathrin-mediated endocytosis by NIMA family kinases
Source: PLoS Genet. 2020 Feb 18;16(2):e1008633. doi: 10.1371/journal.pgen.1008633 (PMC7048319; doi:10.1371/journal.pgen.1008633)
Supplement: Attachment — Submitted filename: PLoS_Response.docx [file pgen.1008633.s028.docx]

We thank the reviewers for their positive comments and suggestions, which we address below. New information in the manuscript is indicated by blue writing. We would also like to note the addition of several experiments not requested by the reviewers but that we feel strengthen the manuscript.

(i) We recently succeeded at generating a NEKL-3::AID strain containing a null mutation in *fcho-1*. Consistent with our previous data showing suppression of NEKL-2::AID clathrin defects with *fcho-1* and *apa-2* (AP2 alpha) mutations, we find that loss of FCHO-1 activity suppresses clathrin defects in auxin-treated NEKL-3::AID worms. Suppression was observed for mean intensity, percent positive pixels, and for clathrin mobility. We now include this result as a new supplementary figure (Lines 433–435. 446–451; S5 Fig).

(ii) We now include data for partial rescue by NEK6::GFP and NEK7::GFP in *nekl-3(gk506)* null mutants (Lines 531–533; S7 Fig).

**Reviewer #1**: Joseph et al. report on a potential endocytic role for the NIMA-like kinases NEKL-2 and NEKL-3. The general function of these kinases has not been well-described, although this group has published several papers reporting the molting defects and suppression of the NEKL phenotypes by CDC42 and related actin regulators. The manuscript largely relies on a genetic analysis of the NEKL phenotype and suppression of the phenotype by reducing the function of AP2 subunits or the AP2 activator FCHO. The manuscript is a genetic tour-de-force: the number of alleles, CRISPR-driven domain mutations, and careful measurement of phenotypic penetrances and backgrounds is impressive. Certainly the degree to which the manuscript uses genetics makes it an excellent candidate for PLoS Genetics. There is very little cell biology and no biochemistry, which makes me somewhat uncomfortable with the specificity of some of the manuscript’s conclusions. It is an absolutely huge manuscript in every respect (10 figures, 8000 word main text, 139 references) – I know PLoS Genetics has no word limit, but some editing for conciseness would make the manuscript much more approachable. Statistics and N numbers appear to be well-done and reported. In the end, I am concerned about some of the reported interpretations, so would like to know more about how specific the AP2 suppression of the NEKL mutants is, as well as additional details of the Clathrin:GFP and LRP:GFP localization.

We have removed a significant portion of the Discussion section, specifically that concerning actin, and have pared down the Introduction, to decrease the size of the manuscript.

1) Perhaps the biggest concern is that the authors strongly tie NEKL function to CME/AP2 due to the functional suppression of the NEKL phenotype. As the authors state, various perturbations of membrane trafficking pathways (for example, even perturbation of ER-Golgi trafficking) can cause molting defects. If NEKL or other molting mutants simply reflect a sensitivity of molting to trafficking defects, I would be concerned about how directly the authors tie NEKL function to clathrin uncoating (a very specific model based largely, but not only, on genetics). To test this, does general disruption of endocytosis suppress NEKL phenotypes, or does decreasing exocytosis (exocyst mutants, secretory Rab mutants) enhance these phenotypes? Would Dynamin mutants produce a similar AP2-like suppression? The NCAP-1 data is interesting, but since it acts in opposition to FCHO-1, these data do not appear to address this issue.

To address this question we first tested two factors, *itsn-1*/intersectin and *ehs-1*/EPS15, which have been shown to physically and functionally interact with FCHO–AP2. We find that rather than suppressing *nekl* molting defects, inhibition of these genes enhances molting defects. Specifically, strains containing loss-of-function mutations in *itsn-1* or *ehs-1* are hypersensitive to partial *nekl–mlt* loss of function. We went on to test several other early-acting endocytosis factors (*unc-11*/PICALM, *unc-57*/endophilin A2, *sel-5*/AAK1, and *lst-4*/SNX9/18/33) and observed enhancement of molting defects in all cases. We have included a description of these findings (lines 589–601; S8 Fig), which indicate that mutations in most genes connected to clathrin-mediated endocytosis enhance rather than suppress *nekl* molting defects. These data indicate that suppression of *nekl* molting defects by reduced AP2 activity is highly specific. These findings are consistent with our data indicating that reduced NEKL activity impairs clathrin-mediated endocytosis. As such, *nekl* phenotypes can apparently be enhanced by inhibiting other endocytic pathway components.

2) Along with the above, the second main part of the argument for NEKL controlling clathrin uncoating are the alterations in clathrin levels and LRP localization. I am sure that there are limitations in the worm system, but the degree of characterization of these defects is somewhat poor. The authors should point out what Clathrin structures they think are aberrantly changing. It appears that there are quite large, intense puncta, which I would be doubtful are true surface endocytic CCPs due to their size. There are other, much less intense, and hard-to-make-out signals that may constitute the major increase in signal in NEKL mutants. Is this true? What are the structural changes? Are the larger (or smaller) puncta Golgi or endosomes? How deep of a focal plane are the authors sampling? A dextran-uptake experiment would be a good addition and would reveal whether endocytic rates are changed, and if the dextran is trapped in the “still caged” clathrin vesicles. Also, does sec-23 cause similar changes in Clathrin localization? Absent some refinement of the data as suggested in points #1 and #2 I would be concerned about how molecularly-specific the authors' model of NEKL function is.

We understand the reviewer’s general points, although we do note some limitations in the *C. elegans* system. For example, dextran will not penetrant the cuticle, so dextran uptake experiments cannot be done in the epidermis. Nevertheless, to address this issue, improve clarity, and strengthen our conclusions, we have made several changes as indicated below.

(i) To help orient readers, we have added new annotated Z-stack movies (Movies S1–6, S13,14) corresponding to eight static figure panels (Fig 6D,G, S2A Fig 10A). These movies indicate the location of the cuticle (alae), the apical-most plane (plasma membrane) of the epidermis, as well as medial planes of the epidermis. Readers can now directly compare differences in the localization of GFP::CHC-1 and LRP-1::GFP between the genotypes at multiple depths.

(ii) To specifically assay for changes in plasma-membrane associated clathrin, we have performed NEKL-3::AID depletion studies in strains doubly marked for clathrin (GFP::CHC-1) and DPY-23/µ2 (mScarlet::DPY-23) (new Fig 7). We find that apical clathrin puncta strongly co-localize with DPY-23/µ2 in both wild-type and NEKL-3::AID depleted worms. This indicates that changes in clathrin localization are occurring at or near the apical plasma membrane and are associated with AP2. In addition, we quantified localization changes for both clathrin and DPY-23/µ2 in the double-marked NEKL-3::AID strain. We observed that both GFP::CHC-1 and mScarlet::DPY-23/µ2 have increased mean intensities and percent pixels above threshold following NEKL-3 depletion. In addition, the extent of co-localization between clathrin and DPY-23/µ2 is increased in NEKL-3-depleted adults, consistent with clathrin accumulating on clathrin-coated pits or vesicles of the AP2 plasma membrane derived type (Lines 365–374; Fig 7). Our findings also suggest that NEKL-3 may affect both clathrin and AP2 uncoating, and is consistent with the idea that AP2 uncoating may facilitate clathrin uncoating in the worm epidermis.

(iii) As a further test, we assayed colocalization between clathrin and a marker for AP1 (mScarlet::APM-1/µ1), which marks clathrin-coated pits and vesicles derived from the trans-golgi network, in auxin-treated wild-type and NEKL-3::AID adults. In contrast with the AP2 marker, co-localization between clathrin and APM-1/µ1 slightly decreased following NEKL-3 depletion, as might be expected if clathrin is overaccumulating at the plasma membrane and less free clathrin is available for TGN associated CCV assembly (Lines 374–378; S3 Fig).

Regarding other types of quantification (e.g., puncta number, size, etc.). We have invested considerable time, both pre-submission and post-submission, into determining if these types of parameters could be reliably assayed in our system. We find that both the process of quantification and the subsequent interpretation of these data is highly challenging and problematic. For example, when quantifying fluorescence images for size and number estimates, thresholding is a vital step. However, both the size and number of puncta can be grossly underrepresented or overrepresented depending on which thresholding algorithms are used, perhaps due to the very high density of clathrin puncta in the hypodermal apical membrane. Performing manual thresholding is somewhat more reliable, however, different areas within the worm epidermis can still be underrepresented or overrepresented, and manual thresholding can also lead to bias. In general, we observed that thresholding was inconsistent with respect to differentiating between multiple puncta that are close together versus single larger puncta. These issues varied from worm-to-worm and also between different regions within individual worms. As such, we cannot not be confident in a key step of the quantification process.

Another issue is that each confocal image has a depth of ~1 μm, which leads to uncertainty regarding the structure(s) one is observing. For example, it is impossible to differentiate between newly forming clathrin pits, pits that are close to pinching off, and apical clathrin-coated vesicles, as well as apical endosomes and Golgi ministacks. The usual technique to answer this question would be to perform TIRF microcopy, which cannot be done in worms due to the presence of the cuticle. We do remain confident in our measurements of mean intensity and percent pixels of above threshold, which provide two non-biased quantification measures for changes in localization.

3) Similarly to the above, please point out the relevant LRP:GFP puncta – the manuscript says these puncta are “likely clathrin-coated pits” or “endosomes”, but this isn’t shown. Can colocalization with Clathrin or AP2 or some CME marker not be done? Even dextran-uptake could partially address this. The degree to which there is inference of compartmental identity based on little-to-no data is concerning.

To address questions regarding LRP-1, we have quantified LRP-1::GFP in medial planes, and show that medial changes are less extensive than those at the apical surface (S6 Fig). We also provide Z-stacks movies of LRP-1::GFP in the wild-type and NEKL-3::AID backgrounds (auxin +), which show that apical defects in LRP-1::GFP localization following NEKL-3::AID depletion are much more dramatic than medial changes (Movies S14,14). Finally, we carried out co-localization of LRP-1::GFP with a marker for AP2 (mScarlet::DPY-23/µ2) in wild-type worms (S6 Fig). This analysis demonstrates that in wild-type adults, LRP-1::GFP localizes extensively with AP2 in apical puncta, consistent with NEKL-3 depletion primarily affecting LRP-1 at or near the plasma membrane. (See lines 462–478.)

4) The NEKL-AID experiments use wild-type as a control – is there a generic AID-containing strain that could be used for a more appropriate control?

We understand the reviewer’s point, but believe that our two main controls, Wild-type +/– auxin, and NEKL::AID +/– auxin are sufficient to show that the defects we see are specific to NEKL reduction. In addition, we have used an epidermal hyp7-specific promoter driven TIR1 to control NEKL levels and have obtained similar or identical results to those reported in the paper.

5) The Discussion, like the rest of the manuscript, is extensive. I am not sure that the connection between CDC42 and clathrin uncoating is appropriate. Although CDC42-driven F-actin certainly has been implicated in scission and early endocytic events, these do not necessarily connect to the uncoating phenotype (or the authors should feel free to suggest more specifics on this).

We agree and have removed this section.

a) As noted above, the manuscript is enormous. It would be improved if it was edited down a bit. The Introduction meandered, and it wasn’t always clear why certain topics were being discussed. It also didn’t seem necessary to include some of the discussed information.

We shortened this section by removing information about the mammalian NEKL orthologs, which are also addressed in the Discussion.

b) Please consider including the relevant mammalian homologs next to the worm gene names throughout, or in many more places – I was constantly going back and needing to look up which endocytic protein a particular worm gene encoded for.

We understand the reviewer’s point and have added information about the identity of the AP2 subunits, where possible, throughout the manuscript.

**Reviewer #2:** This work corresponds to a follow-up study of the role and positioning of the NIMA family kinases which the Fay lab had initially characterized. Here the authors position the NEKL-2/3 proteins within the endocytic pathway by showing (i) that nekl-2; nekl-3 lof molting defects can be suppressed by mutations affecting AP-2 subunits of proteins required for AP-2 activation; (ii) conversely that mutations in proteins favoring AP-2 closed conformation enhance the phenotype of a weal nekl-2 or nekl-3; (iii) that nekl-2: nekl-3 loss affects clathrin recycling and induces plasma an accumulation of lipoprotein LRP-1 in vesicles or the plasma membrane apically. The authors conclude that NEKL-2/3 act in the endocytic pathway to favor through a process that remains to be characterized at the molecular level AP-2 closed conformation, perhaps by favoring clathrin uncoating from internalized vesicles.

I enjoyed reading this very well documented and written manuscript. I believe that it should be of interest to a wide audience and I therefore enthusiastically recommend for publication once some issues have been solved.

The main issue that the authors should address is to perform one more experiment to test their prediction regarding the role of NEKLs in uncoating clathrin from internalized coated vesicles. This would require EM (which they mention) to define is uncoated vesicles accumulate, or probably easier to implement examine the genetic interactions between nekl-2/3 lof and auxilin and/or Hsc70.

We currently have no EM data addressing the structure/appearance of clathrin-coated pits in the worm epidermis, although we are now collaborating with another group to (hopefully) obtain this kind of information in the future, but this is beyond the scope of the current manuscript. So far as we are aware, no previous studies have visualized clathrin pits using EM in the worm epidermis.

As suggested, however, we have tested for genetic interactions between the *nekls* and one of the two auxilin/cyclin G-associated kinase homologs in *C. elegans*, *dnj-25*. *C. elegans* *dnj-25* has five isoforms, which can be grouped into the long (a1, a2, d1) and short (b1, c1) variants. We first carried out two sets of RNAi injections using regions that targeted either the long isoforms specifically, or all five isoforms. Surprisingly, we failed to detect any molting phenotype, which would have been expected based on a previous report. We note that we used exon-rich genomic regions rather than a cDNA as our starting template for dsRNA synthesis, which could have reduced RNAi efficacy to some extent. However, we also failed to observe enhancement of molting defects when these dsRNAs were injected into *nekl-2/3* hypomorphs, which was surprising.

We next turned to CRISPR to make mutations affecting the long isoforms or all five isoforms. We obtained several strains that should specifically abolish activity of the long isoforms and observed an absence of molting defects, consistent with our RNAi data. Furthermore, these strains were not hypersensitive to partial inhibition of *nekls* using RNAi feeding, also consistent with our RNAi data. We have tried but thus far failed to isolate a CRISPR mutation that affects all five isoforms.

We have also not obtained clear results yet with *hsp-1*/HSPA8/Hsc70 mutants/RNAi. Thus, at the present time, we have no definitive data regarding genetic interactions between *nekls* and auxilin/Hsc70 and this will clearly take more time to sort out. In addition, we will also likely need to test the role of a cyclin G-associated kinase homolog, *gakh-1*. Given the large amount of data already in the paper and our efforts thus far, we feel it would be reasonable to set aside investigations between the NEKLs, auxilin /Hsc70, and clathrin uncoating, for a future study.

Another recommendation would be to speculate on the potential molecular target of NEKLs and avenues to test them. If needed the discussion on the actin phenotype of nekl-2/3 mutants could be trimmed down since actin is not brought up in the results but was part of a previous study. Of note, some v-ATPase subunits also undergo a complete reorganization during molting.

We understand the interest in knowing the NEKL kinase targets. But given the length of the current manuscript (also see comments for reviewer #1), we are hesitant to speculate on possible targets of the NEKLs at this time. We hope to be able to address this question more definitively in future papers.

Minor points:
- Please number pages

We have now numbered the pages and lines.

- Please make a separate supplementary table of all strains (it is currently very difficult to read in the M&M)

We have now added a new table (S2 Table) containing this information.

- Consider reducing the number of references (nice to have a complete listing, but it takes room – I leave it to the Editor to decide)

As also suggested by reviewer #1, we have reduced the size of the manuscript, including the number of rotations.

- An s missing at the very top of page 20 (in the PDF downloaded from the PLoS website) in “statically“

This has been fixed.

- In movie S4, the recovery after FRAP does not appear to be homogeneous since there are clear dots appearing in a background of slow recovery, which I didn’t spot in other conditions. Could this tell us something about NEKL-2 function?

This is a good observation. We have re-examined worms used for the FRAP quantifications in NEKL-2::AID and NEKL-3::AID auxin treated samples. We observed that both NEKL-2::AID and NEKl-3::AID auxin-treated worms exhibited a mix of homogenous and nonhomogeneous recovery. However, nonhomogeneous recovery was observed more frequently in NEKL-2::AID worms. We now state, “We note that some bleached spots exhibited homogenous recovery within the bleached area whereas others exhibited nonhomogeneous recovery. This was true for both NEKL-2::AID and NEKl-3::AID auxin-treated worms, although nonhomogeneous recovery was observed more frequently in NEKL-2::AID (16/21) than in NEKL-3::AID (5/17) samples. We used movies and images for FRAP that were most representative. (Lines 409–413.)

Reviewer #3: The manuscript by Joseph et al describes the role of the NEKL kinases in regulating endocytosis in the C. elegans epidermis. They first use a lethal phenotype to demonstrate the genetic interactions between nekl-2/-3 and AP2 subunits or AP2 regulators (fcho-1 and ncap-1), using in particular precise gene editing by CRISPR/Cas9; the results are then confirmed with the analysis of the Jowls phenotype. These genetic interactions are further confirmed by examining the localisation of an endogenous reporter for clathrin and an auxin-based degron system. To better understand how NEKL kinases control clathrin dynamics and endocytosis they next use FRAP experiments and examine the localisation of LRP-1, an apical transmembrane protein. They conclude that NEKL kinases regulates an AP2 dependent trafficking step. Finally they show that human homologs can also be implicated in trafficking.

This a very well written manuscript, easy to follow, and most experiments are carefully done and analyzed. In particular the use of gene editing and endogenously expressed markers makes the results quite convincing. The conclusions of the study are well supported by the experimental data.

I only have a few points to raise:

1) The most important one is the quantification of clathrin and LRP-1 localisation. A punctate staining can be tricky to analyze based solely on mean intensity and % of positive pixels, as you can in theory get similar results with very different patterns. If some results are quite convincing (eg Fig5), others are less obvious (Fig9C and Fig10A). For instance in Fig 10 the authors found no difference in clathrin localisation while the pictures they show look different between control and the ncap-1;dpy-23 double mutant. I recommend that the authors use a more detailed quantification method, analyzing the number of puncta, their intensity and their size in all ambiguous situations as this could reveal more subtle changes.

We understand the reviewer’s point. As discussed above in our response to reviewer #1 (point #2), we have attempted to measure some of these parameters in our system, and have concluded that we cannot obtain what we consider to be reliable information at this time. With respect to differences between some of the panels (e.g., new Fig 11A), we find that there is considerable variability between individual worms within a given genotype. We chose representative images for panels based in large part on them having close to average quantification values (for MI and PP). As for differences in the appearance of GFP::CHC-1 between panels (e.g., Fig 11A), we observed (by eye) what may be a slight trend towards *ncap-1; dpy-23* mutants having somewhat more small puncta and somewhat fewer large puncta than wild type, which is partly why the specific panel was chosen – because in our view it was most representative of the sample population. Admittedly, this is somewhat subjective. We have edited our text to state “Likewise, we failed to detect statistical differences in clathrin localization in *ncap-1(mew39)*; *dpy-23(mew74)* double mutants, although there appeared to be a trend towards a reduced number of the larger clathrin puncta and an increased number of smaller puncta in this strain (Fig 11A–C; Lines 496–499).

2) While the expression pattern of NEKL-2 and NEKL-3 has been described by the same group previously (Lažetić and Fay, 2017), the authors do not attempt to colocalize these proteins with either clathrin or AP2 subunits. Given their conclusions on NEKL-2/-3 possible function, it would be interesting to observe a possible colocalization.

This is a reasonable point and an interesting question. Using our CRISPR NEKL alleles, we have found that subapical and medial NEKL-3 strongly co-localizes with the early endosome marker, RAB-5. Likewise, subapical/medial NEKL-2 co-localizes quite extensively with the late endosome marker, RAB-7, but also to some extent with RAB-5. Although we do see NEKL-2/3 expression at or near the apical surface (both diffuse and in puncta), we have not observed convincing co-localization of NEKLs at clathrin-coated pits. We note, however, that Rab5, which is known to localize to clathrin-coated pits as well as vesicles and early endosomes, can also be difficult to detect at the plasma membrane as levels tend to be quite low. Auxilin and GAK are also very difficult to colocalize to clathrin-coated pits, since Auxilin/GAK only appear on pits in a burst during the last 5 seconds of a 1-2 minute coated pit formation process, with 4-5 times fewer molecules of auxilin than clathrin in the pit (Massol 2006 PMID: 16798879; Lee 2006 PMID: 16895969). Thus, NEKLs could also be acting (catalytically) at the apical surface, but at relatively low levels that are difficult to capture. We would prefer not to include the current co-localization studies as we are uncertain about what these data is telling us and feel that it’s inclusion would be premature. In addition, as pointed out, the manuscript is already very long.

3) FRAP controls look very similar in Fig 7F and Fig 10D-E. If they are identical it should be mentioned, and the reason for using the same control justified in the M&M section.

The reviewer is correct. FRAP data acquired for Figures 7 and 10 (now 8 and 11) were carried out at the same time using the same scope and acquisition procedures. For this reason, some of the same control data is presented in these figures. We have how indicated this in the M&M section (Lines 778–780).

Minor points:
Remove a ; in the CRISPR § of the M&M section.

This has been done.

Briefly describe the western blot conditions in M&M

This has been done.

As far as I am aware the original apb-1 loss of function phenotype was first described by Shim et al 2000 in an MBoC paper which could be added to the references.

This has been done.
